# Hsa-miR-107 regulates chemosensitivity and inhibits tumor growth in hepatocellular carcinoma cells

**DOI:** 10.18632/aging.202908

**Published:** 2021-04-26

**Authors:** Hsin-An Chen, Chi-Cheng Li, Yu-Jung Lin, Tso-Fu Wang, Ming-Cheng Chen, Yen-Hao Su, Yu-Lan Yeh, V. Vijaya Padma, Po-Hsiang Liao, Chih-Yang Huang

**Affiliations:** 1 Graduate Institute of Clinical Medicine, College of Medicine, Taipei Medical University, Taipei City 250, Taiwan; 2 Department of Surgery, School of Medicine, College of Medicine, Taipei Medical University, Taipei City 250, Taiwan; 3 Division of General Surgery, Department of Surgery, Shuang Ho Hospital, Taipei Medical University, New Taipei City 235, Taiwan; 4 TMU Research Center of Cancer Translational Medicine, Taipei Medical University, Taipei City 250, Taiwan; 5 Center of Stem Cell & Precision Medicine, Hualien Tzu Chi Hospital, Buddhist Tzu Chi Medical Foundation, Hualien 970, Taiwan; 6 Cardiovascular and Mitochondrial Related Disease Research Center, Hualien Tzu Chi Hospital, Buddhist Tzu Chi Medical Foundation, Hualien 970, Taiwan; 7 Department of Hematology and Oncology, Hualien Tzu Chi Hospital, Buddhist Tzu Chi Medical Foundation, Hualien 970, Taiwan; 8 Division of Colorectal Surgery, Department of Surgery, Taichung Veterans General Hospital, Taichung 407, Taiwan; 9 Department of Pathology, Changhua Christian Hospital, Changhua 500, Taiwan; 10 Department of Medical Technology, Jen-Teh Junior College of Medicine, Nursing and Management, Miaoli 356, Taiwan; 11 Department of Biotechnology, Bharathiar University, Coimbatore 641046, India; 12 Center of General Education, Buddhist Tzu Chi Medical Foundation, Tzu Chi University of Science and Technology, Hualien 970, Taiwan; 13 Department of Medical Research, China Medical University Hospital, China Medical University, Taichung 404, Taiwan; 14 Graduate Institute of Biomedical Sciences, China Medical University, Taichung 404, Taiwan; 15 Department of Medical Laboratory Science and Biotechnology, Asia University, Taichung 413, Taiwan

**Keywords:** chemosensitivity, miR-107, hepatocellular carcinoma, drug resistance

## Abstract

Hepatocellular carcinoma is a common type of liver cancer. Resistance to chemotherapeutic agents is a major problem in cancer therapy. MicroRNAs have been reported in cancer development and tumor growth; however, the relationship between chemoresistance and hepatocellular carcinoma needs to be fully investigated. Here, we treated hepatocellular carcinoma cell line (HA22T) with a histone deacetylase inhibitor to establish hepatocellular carcinoma-resistant cells (HDACi-R) and investigated the molecular mechanisms of chemoresistance in HCC cells. Although histone deacetylase inhibitor could not enhance cell death in HDACi-R but upregulation of miR-107 decreased cell viability both in parental cells and resistance cells, decreased the expression of cofilin-1, enhanced ROS-induced cell apoptosis, and dose-dependently sensitized HDACi-R to HDACi. Further, miR-107 upregulation resulted in tumor cell disorganization in both HA22T and HDACi-R in a mice xenograft model. Our findings demonstrated that miR-107 downregulation leads to hepatocellular carcinoma cell resistance in HDACi via a cofilin-1-dependent molecular mechanism and ROS accumulation.

## INTRODUCTION

Hepatocellular carcinoma (HCC) is a serious health issue worldwide as a primary malignancy and is the third leading cause of cancer-related deaths in the world [1]. The pathophysiology of HCC continues to be studied. Hepatitis B infection and other etiologies underlying cirrhosis are highly associated with HCC [2, 3]. Recently, some studies have shown that metabolic syndrome and repeated inflammation enhance carcinogenesis [4, 5]. Together, these findings indicate that HCC mainly occurs in the liver with cirrhosis, repeated inflammation, and fibrogenesis.

Currently, there are three important therapeutic approaches used in HCC therapy such as surgery, radiation therapy, and chemotherapy. Primary tumor (no metastasis) can usually be removed by surgery such as hepatectomy and liver transplantation [6]. However, liver transplantation has shown no significant improvement in survival rate and prognosis in patients with HCC [7]. Moreover, targeted therapy, a novel type of chemotherapy, involves drugs inhibiting cancer-specific molecules such as vascular endothelial growth factor (VEGF) and histone deacetylases (HDAC) [8–10]. Importantly, some clinical cases show a failure of chemotherapeutic drugs to inhibit tumor growth in patients or after a few treatments, indicating that the tumors become chemoresistant [11]. Chemoresistance results in relapse after few cycles of chemotherapy or targeted therapy. There are two types of chemoresistance, one is present before therapy named primary resistance, and the other one is acquired resistance, which occurs during treatment cycles [12, 13].

Cofilin-1 and other proteins with similar biological functions belong to the actin depolymerizing factor (ADF) family. [14]. After activation by dephosphorylation by phosphatases, cofilin-1 interacts with actin and promotes actin remodeling to promote cell migration [15]. Previous studies have identified that the mechanism of cofilin-1 induces cell apoptosis is translocate to mitochondria [16–18] and only the active form of cofilin-1 will translocate mitochondria [19]. Other studies also reported that treat with reactive oxygen species (ROS) inducer results in mitochondria dysfunction through cofilin-1 in liver cancer cells [20] and activated cofilin interacts with Bcl-2-associated X protein (BAX) and translocates to the mitochondria to promote neuronal cell apoptosis [16]. Importantly, our previous research demonstrated that chemotherapeutic drug-induced HCC cell death via activation of cofilin-1 is related to interaction with BAX and ROS accumulation. Phosphorylation of cofilin-1 can thus lead to HCC resistance to chemotherapeutic drugs [21].

MicroRNAs (miRNAs) are approximately 20–22 nucleotides of short RNA [22]. They bind to the 3' untranslated region (3' UTR) on the target mRNAs, which may have perfect complementarity that would result in mRNA degradation or imperfect complementarity to inhibit mRNA translation or subsequent reduction in protein expression. The molecular mechanism of miRNAs in tumor such as pathogenesis, and the therapeutic response had been demonstrated, and these miRNAs have been reported as potential biomarkers in cancer [23–30]. Although there are many studies that show that miRNAs play important roles in tumorigenesis, their role in liver cancer resistance to chemotherapeutic drugs and the miRNA transfer in cell cross-talks need to be fully elucidated.

Our previous study identified a potential mechanism of drug resistance in liver cancer. In this study, we focus on the role of the miRNAs between parental and drug-resistant cells to reveal potential therapeutic targets in liver cancer.

## RESULTS

### Expression of miR-107 decreased in HDACi-resistance HCC cells compared to parental cells

**Figure 1:**
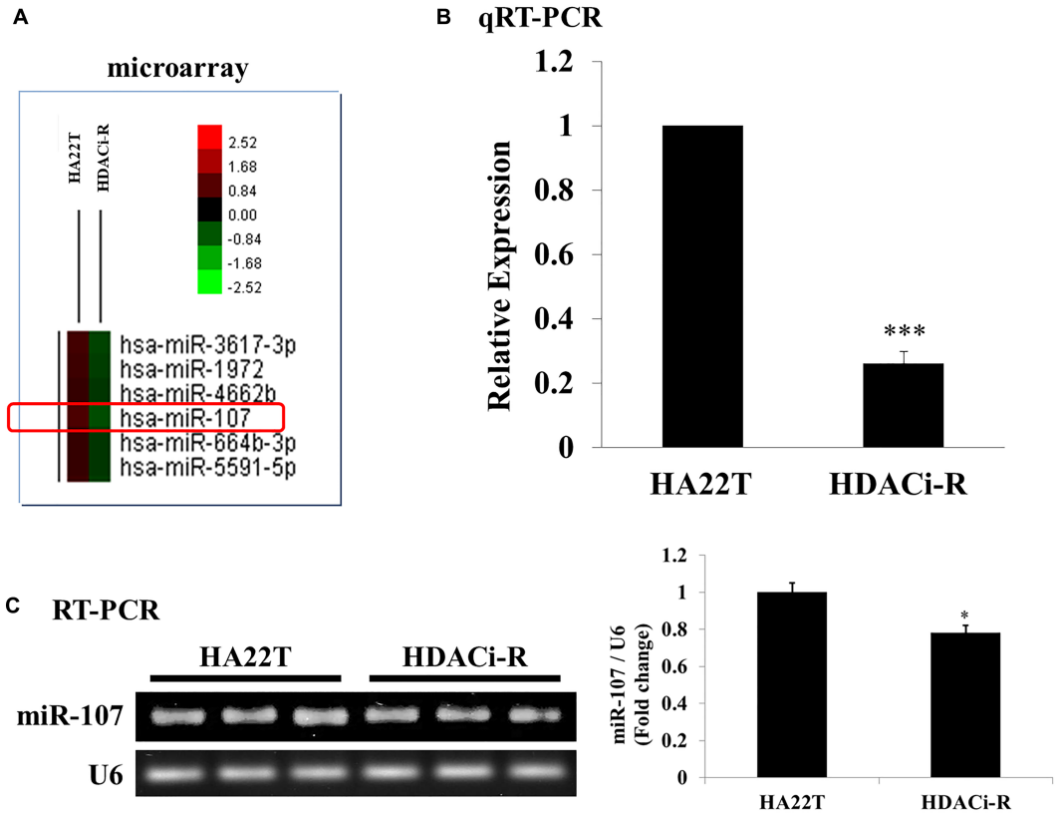
**Expression of microRNA-107 (miR-107) in HA22T and HDACi-R.** (**A**) Detection and comparison of expression of miRNAs between HA22T and HDACi-R (histone deacetylase inhibitor-resistant) cells by microarray assay. (**B**) Confirmation of decrease in expression of miR-107 in HDACi-R cells by quantitative RT-PCR. (**C**) Double confirmation of decrease in expression of miR-107 in HDACi-R cells by RT-PCR.

In our previous study [21] we established HDACi (HDAC inhibitor)-R cell lines by challenging HA22T cells with HDAC inhibitors (apicidin and SAHA) to. Here, we used HA22T and HDACi-R cell lines to investigate the role of miRNAs in drug resistance in HCC. We chose miR-107, which has been implicated in the regulation of cofilin-1 expression based on previous findings and the microRNA.org database. Figure 1A is a microarray heatmap that shows that miRNA-107 expression (HDACi-R/HA22T log2 ratio = -1.640506638; *p* values = 0.004507765) decreased in HDACi-resistant cells (HDACi-R) compared to that in parental cells (HA22T). Next, we confirmed that the expression of miR-107 in resistant cells was indeed lower than that in parental cells by qRT-PCR (Figure 1B) and RT-PCR (Figure 1C). These data suggest that expression of miR-107 was downregulated in resistant cells.

### Relationship between miR-107 and chemosensitivity in HCC cells

**Figure 2:**
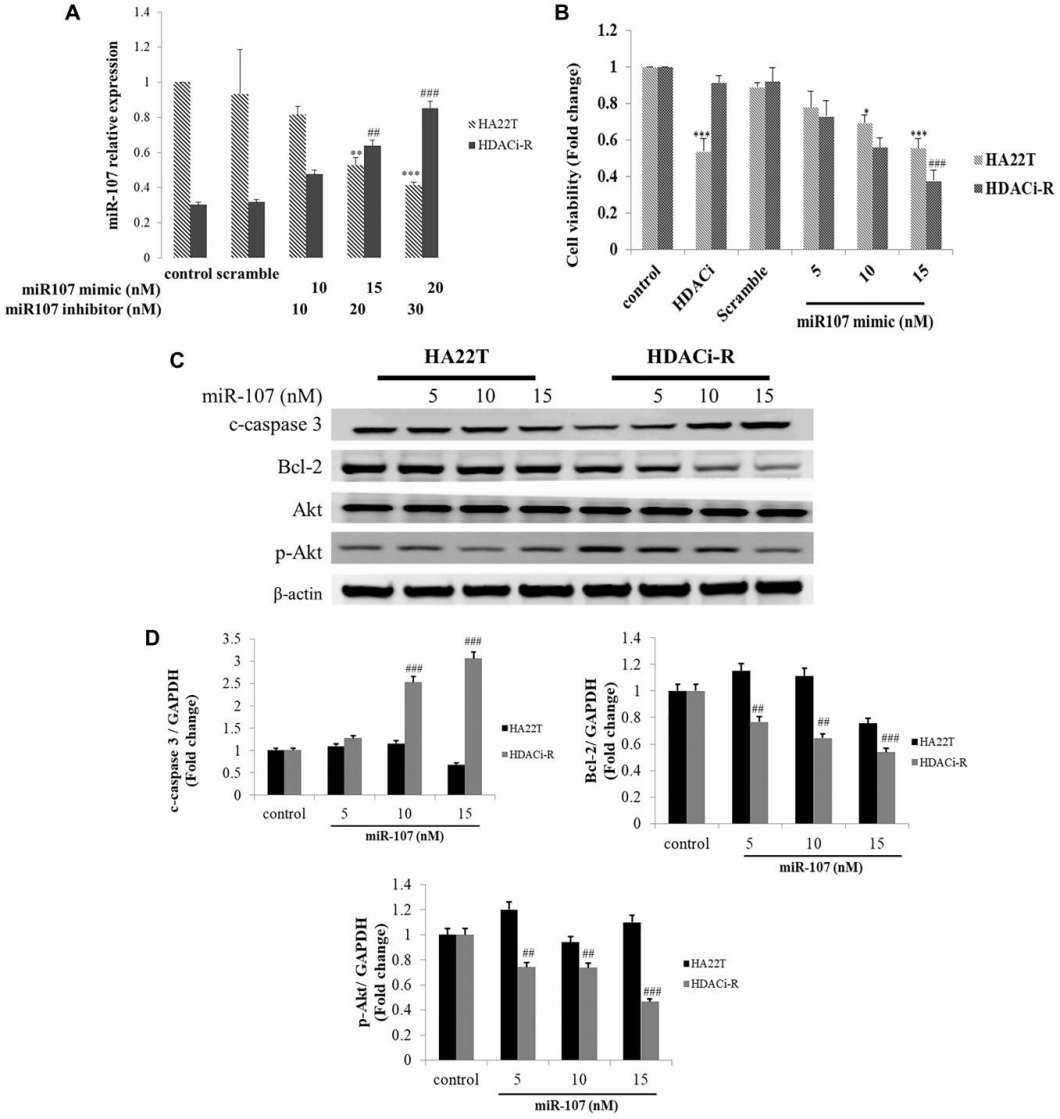
**MicroRNA-107 (miR-107) decreased cell survival and induced cell death in hepatocellular carcinoma (HCC) cells.** (**A**) Regulation of miR-107 expression by transfection with mimic and inhibitor in HA22T and HDACi-R (histone deacetylase inhibitor-resistant) cells detected by quantitative RT-PCR. (**B**) MiR-107 decreased cell viability in HA22T and HDACi-R cells, as assessed by MTT (3-(4,5-dimethylthiazol-2-yl)-2,5-diphenyl tetrazolium bromide) assay. (**C**) MiR-107 promoted apoptosis-related proteins such as cleaved caspase-3 and decreased expression of pro-survival-related proteins. (**D**) Quantification of Figure 2C. Expression of fold change of cleavage caspase-3, Bcl-2, and p-Akt after normalization using actin. ^*^P < 0.05, ^***^P < 0.001 vs. HA22T control. ^#^*P* < 0.05, ^###^*P* < 0.001 vs. HDACi-R control.

Figure 1 shows that the expression of miR-107 was downregulated in HDACi-resistant cells. Following this, we determined the role of miR-107 in chemoresistance in two HCC cells using transfected miR-107 mimic and miR-107 antisense (inhibitor). Initially, we examined whether miR-107 expression changes after transfection of HDACi-R and HA22T cells with a mimic or an inhibitor of miR107, respectively. Figure 2A shows a lower miR-107 expression in HDACi-R compared to HA22T in control, untransfected cells. The expression of miR-107 was upregulated after transfection with the mimic in HDACi-R cells and was downregulated after knockdown of miR-107 using transfection of inhibitor in parental cells in a dose-dependent manner. Further, we investigated whether miR-107 induces HCC cell apoptosis. The results of the MTT assay showed that HDACi induced cell death only in HA22T cells, and that upregulation of miR-107 in HA22T and HDACi-R cells decreased cell viability (Figure 2B). Moreover, we observed that the upregulation of miRNA could induce apoptosis-related proteins such as cleaved caspase-3 (c-caspase-3) and decrease pro-survival-related proteins such as Bcl-2 and p-Akt expression in HDACi-R and HA22T cells, as confirmed by western blotting analysis (Figure 2C) and quantification (Figure 2D). Importantly, Figure 2B–2D show that miR-107 regulated cell viability and expression of apoptosis-related and pro-survival-related proteins, especially in HDACi-R cells. Interestingly, comparing the protein basal levels of the two cell lines, resistant cells have a lower caspase 3 and Bcl-2 and a higher p-Akt expression, compared to HA22T. We also noticed that overexpressed miR-107 decreased cell viability both in two cell lines (Figure 2B) hint that miR-107 may induced cell death both in two cells. However, Figure 2C showed that miR-107 significant increased c-caspase-3 expression and decreased Bcl-2/ p-Akt expression only in HDACi-R cells. These findings indicate that miR-107 plays an important role to regulate cell death and chemoresistance in HCC cells, especially in HDACi-R.

### MiR-107 regulates chemosensitivity in HCC cells

**Figure 3:**
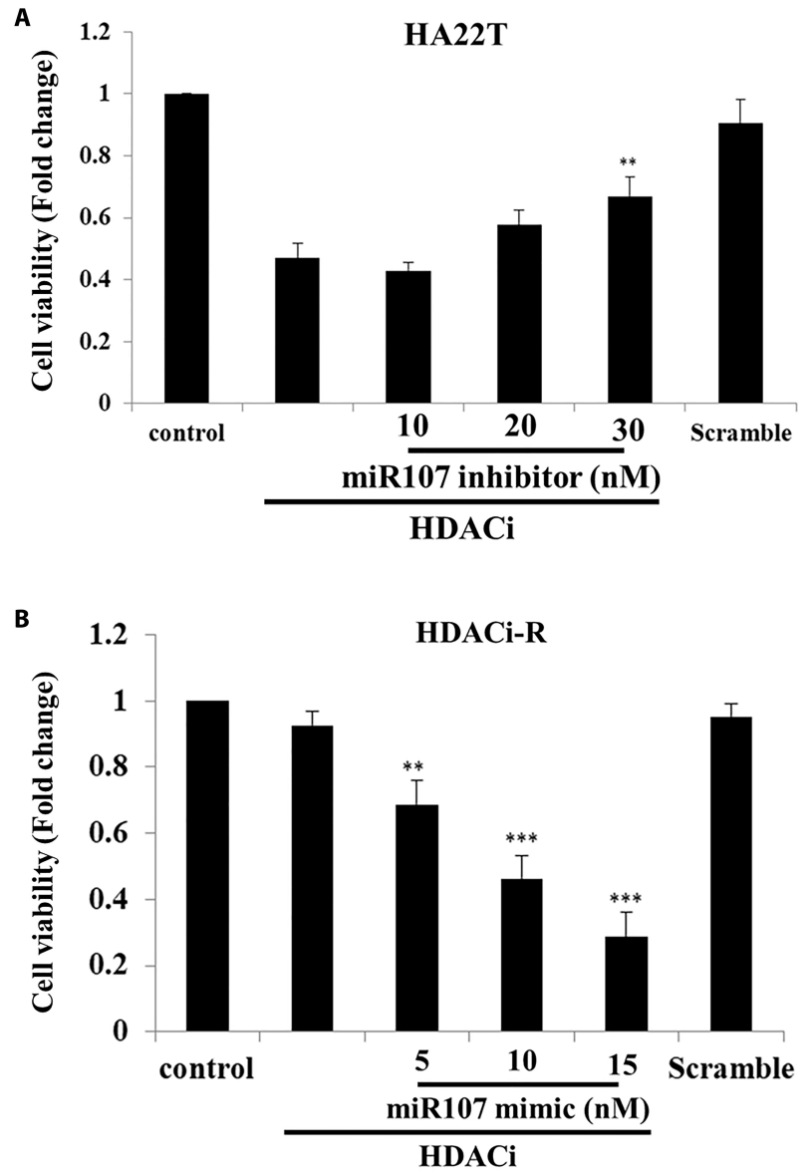
**MicroRNA-107 (miR-107) enhanced chemosensitivity in hepatocellular carcinoma (HCC) cells.** (**A**) Knockdown of expression of miR-107 prevented HDACi (histone deacetylase inhibitor)-induced downregulation of cell viability in HA22T cells, as assessed with MTT (3-(4,5-dimethylthiazol-2-yl)-2,5-diphenyl tetrazolium bromide) assay. (**B**) Upregulation of miR-107 enhanced chemosensitivity in HDACi-R cells, as assessed with MTT assay. Scramble miRNA group treated with 15 μM.

According to our previous results, expression of miR-107 downregulated pro-survival proteins expression in HDACi-R cells, and the upregulation of miR-107 led to increased apoptosis in HDACi-R HCC cells compared to that in parental cells. Next, we determined whether expression of miR-107 affects chemosensitivity in two liver cell lines. In Figure 3A, the MTT assay results indicated that HDACi (SAHA) decreased HA22T cell viability but knockdown of expression of miR-107 rescued HDACi-induced cell death in HA22T cells. Moreover, we confirmed that HDACi cannot induce cell death in HDACi-R cells but upregulation of miR-107 induced HDACi-R cellular sensitivity to HDACi in a dose-dependent manner (Figure 3B). These results indicate that miR-107 is highly correlated with chemosensitivity in HCC cells.

### MiR-107 induced ROS accumulation and cell apoptosis by targeting cofilin-1 in HCC cells

**Figure 4:**
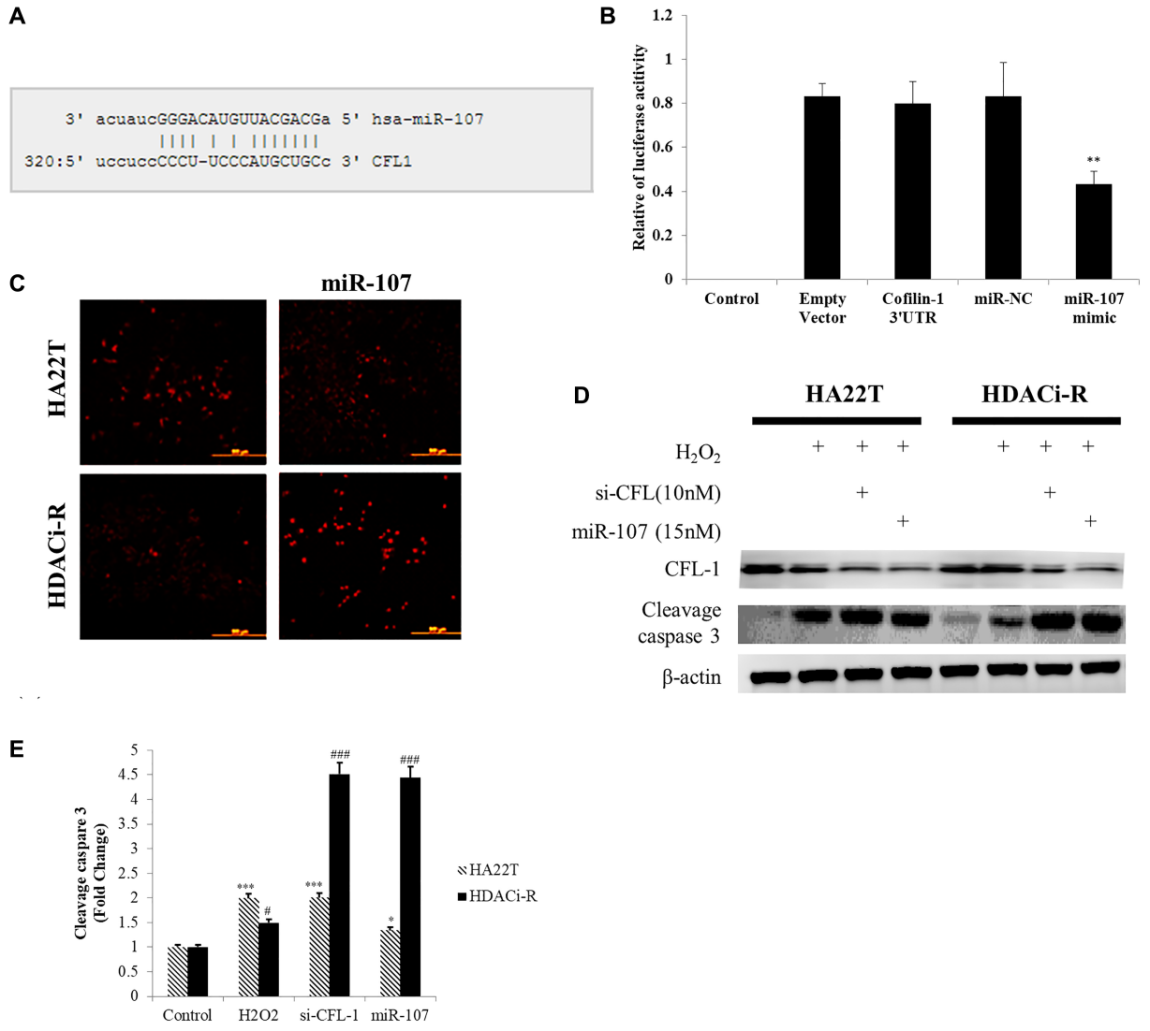
**MicroRNA-107 (miR-107) induced reactive oxygen species (ROS) accumulation and cell death by targeting cofilin-1 in hepatocellular carcinoma (HCC) cells.** (**A**) MiR-107 target sequence on cofilin-1 (CFL1) 3' UTR (untranslated region). (**B**) Luciferase activity assays of luciferase vectors with cofilin-1 3' UTR were performed following transfection with miR-107 or negative control for 24 h. ^**^P < 0.01 vs. HDACi-R (histone deacetylase inhibitor-resistant) cells cofilin-1 3' UTR group. (**C**) MiR-107 induced reactive oxygen species (ROS) accumulation, as detected by MitoSOX staining. (**D**) H_2_O_2_-induced HA22T cell death was higher than HDACi-R cells. Transfection with si-cofilin-1 (si-CFL-1) and miR-107 mimic decreased cofilin-1 (CFL-1) expression and enhanced H_2_O_2_-induced cleavage caspase-3 expression, as detected by western blotting assay. (**E**) Quantification of Figure 4D. Fold change of cleavage caspase-3 after normalization using actin. ^*^P < 0.05, ^***^P < 0.001 vs. HA22T control. ^#^*P* < 0.05, ^###^*P* < 0.001 vs. HDACi-R control.

Previous research has indicated that cofilin-1 leads to chemoresistance by affecting ROS accumulation in HCC cells [21]. Moreover, other studies have indicated that cofilin-1 is a direct target for miR-107 [31] in brain diseases. We then determined whether miR-107 induced cell death by regulating cofilin-1 expression and ROS accumulation. As shown in Figure 4A (predicted binding sites on microrna.org online database) and Figure 4B, the miR-107 binding sequence is located on the cofilin-1 3' UTR, as predicted by mircoRNA.org database. After co-transfecting HDACi-R cells with the cofilin-1 3' UTR reporter vector and miR-107 mimic, luciferase activity was found to be significantly reduced compared with the cofilin-1 3' UTR reporter vector alone. Next, we determined whether cell death induction by miR-107 was related to ROS accumulation. First, we observed that ROS accumulated in HA22T control group more than HDACi-R control group. Upregulated expression of miR-107 induced ROS accumulation in HDACi-R cells (Figure 4C). As presented in Figure 4D, ROS induced higher apoptosis- related protein expression (c-caspase-3, Figure 4E) in HA22T cells than HDACi-R cells. Further, upregulation of miR-107 decreased expression of cofilin-1 and enhanced ROS-induced cell apoptosis. We observed the same result after knockdown of cofilin-1 by siRNA also enhanced HDACi-R sensitivity to ROS (Figure 4D). Importantly, upregulation of miR-107 promoted ROS accumulation in both types of cells, especially in HDACi-R cells. These findings suggest that miR-107 inhibited cell viability through cofilin-1-mediated ROS accumulation in HCC cells.

### MiR-107 inhibits HCC tumor growth in nude mice model

**Figure 5:**
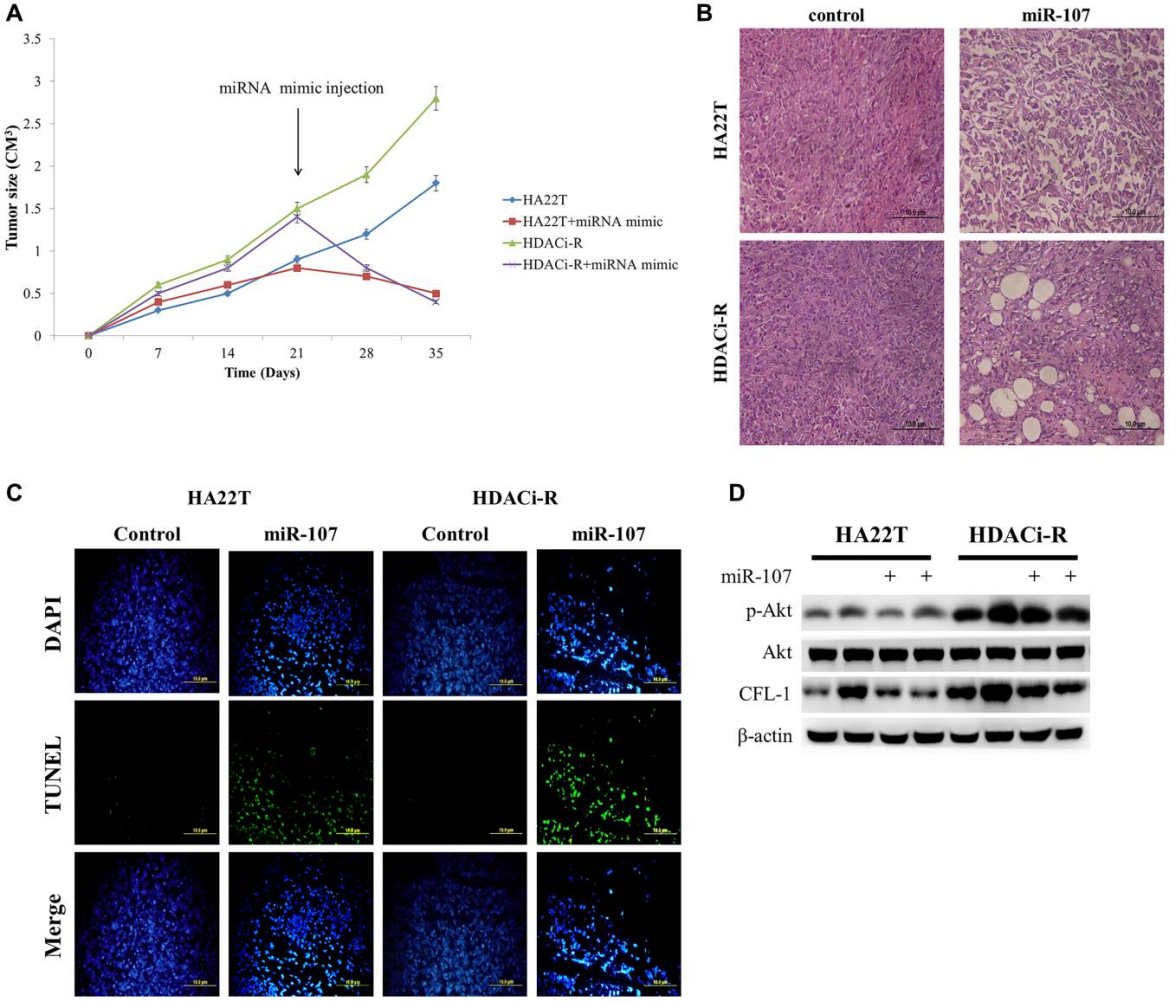
**MicroRNA-107 (miR-107) regulates tumor growth and tumor death in hepatocellular carcinoma (HCC) tumors *in vivo*.** (**A**) The tumoral growth of HCC cell lines xenografted on nude mice. Treatment was administered at day 21 by tumor injection. (**B**) Hematoxylin and eosin (H&E)-stained sections from the xenograft. Scale bar: 100 μm. (**C**) TUNEL assay was performed to visualize apoptotic cells (green), and DAPI staining showed the number of nuclei. Scale bar: 100 μm. (**D**) Overexpression of miR-107 caused a decrease in CFL-1 and p-Akt expression both in two tumors as observed by western blotting assay.

After verification of the role of miR-107 in two HCC cells, we confirmed whether miR-107 regulates HCC tumor growth in a nude mice model. HDACi-R tumor growth was faster than that of HA22T. After tumors were injected with miR-107, we observed significant inhibition of tumor growth, especially in HDACi-R cells (Figure 5A). Histological analysis of tissues sections by hematoxylin and eosin (H&E) staining showed that upregulation of miR-107 resulted in tumor cell disorganization and loss of nucleus in both HA22T and HDACi-R tumors by histological analysis (Figure 5B). These data indicate that miR-107 not only inhibited tumor growth but also induced tumor apoptosis. Next, we used TUNEL (terminal deoxynucleotidyl transferase dUTP nick end labeling) assay to double confirm that miR-107 induced tumor apoptosis. Figure 5C shows that upregulation of miR-107 induced cell apoptosis both in HA22T and HDACi-R tumors. Moreover, we also obtain that upregulation of miR-107 decreased survival protein (p-Akt) and CFL-1 expression both in two HCC tumors (Figure 5D). These findings indicate that miR-107 had an anti-tumor effect and in HCC.

## DISCUSSION

As is well known, drug resistance is an important issue in cancer therapy, as it can lead to treatment failure and poor prognosis. Therefore, we aimed to identify a novel therapeutic target against chemoresistance in HCC. Cofilin-1 is highly correlated with cell metastasis and invasion [32–34], can cause cytoskeleton remodeling [35], and is involved in cell differentiation [36]. These molecular mechanisms indicated that cofilin-1 which is very important gene to regulate cancer cells metastasis. Our previous study [21] indicated that cofilin-1 is a novel chemoresistance-related gene in HCC. Treatment with anti-cancer drug induced ROS accumulation and cell death by promoted cofilin-1 translocation to mitochondria. Accordingly, we revealed a mechanism of drug resistance that involves the ERK phosphorylate cofilin-1, which leads to the decrease in cofilin-1 translocation to the mitochondria, and ROS accumulation, with consequent cell death. These findings suggest that cofilin-1 should be considered a novel therapeutic target against drug resistance in HCC. We also observed that miR-107 enhanced ROS accumulation in resistance cells (Figure 4C); thus, the molecular mechanism of ROS production by miR-107 may involve the regulation of cofilin-1 expression.

Epigenetic regulation such as dysfunctional miRNA expression has been implicated in many cancers [37, 38]. The miRNA expression change is also correlated with the pathogenesis of multiple cancers such as in the lungs, pancreas, and liver to regulate cancer cells initiation, development, and metastasis [39]. Previous studies have indicated that miR-107 is a tumor suppressor in many cancers such as lung cancer [40], melanoma [41], and oral squamous cell carcinoma [42]. However, other studies have shown that miR-107 can induce chemoresistance in colon cancer by targeting calcium-related proteins [43]. Further, miR-107 reportedly promotes HCC cell proliferation by targeting axin2 [44]. Importantly, some reports showed that miR-107 plays an important role in regulating chemosensitivity through its targets in breast cancer [45, 46]. Although these findings elucidated the roles of miR-107, the molecular mechanism of chemoresistance in HCC remains unknown. In this study, we identified that miR-107 is an anti-drug resistance gene in HCC. After establishing chemoresistant cells (HDACi-R cells), we observed that the expression of miR-107 decreased significantly compared with that in parental cells (HA22T). Following this, we found that upregulation of miR-107 in the two types of HCC cells led to cell apoptosis and inhibited tumor growth, especially in HDACi-R group. Moreover, upregulation of miR-107 increased HDACi-R cell sensitivity to HDACi. These results indicate that miR-107 is not only a tumor suppressor gene but is also correlated with chemosensitivity. We also identified that miR-107 induced cancer cell death and increased chemosensitivity by targeting cofilin-1.

Taken together, our study revealed that miR-107 expression was downregulated in drug resistant HCC cell line and the upregulation of miR-107 induced cell death via ROS accumulation and inhibited tumor growth in part through regulating the expression of cofilin-1. These findings present miR-107 as a potential chemosensitizer and drug resistance therapeutic target in patients with HCC.

## MATERIALS AND METHODS

### Cell culture

Liver cancer cell line (HA22T) was purchased from BCRC (Bioresource Collection and Research Centre, Hsinchu, Taiwan). Cells were cultured in Dulbecco’s minimum essential medium (Sigma, St. Louis, Missouri, USA) as previously described [21]. The HDACi-resistant cells resistant to SAHA and apicidin were established from HA22T cells [21]. In this study, the HDACi cells were treated with SAHA (2 μM).

### Whole-cell extraction

The whole-cell protein sample extracted by RIPA Lysis Buffer (Thermo Scientific, USA) with proteinase K and phosphatase inhibitors, as previously described [21, 47]. Briefly, the cell pellets were lysed centrifuged at 12,000 rpm for 15 min at 4°C. The liquid supernatant was collected and stored at -20°C.

### MTT assay

Cell viability was carried out as previously described [21]. 1 × 10^6^ cells were seeded on 24-well plates. MTT reagent (3-(4, 5-dimethylthiazol-2-yl)-2, 5-diphenyltetrazolium bromide; Sigma-Aldrich Inc., St Louis, MO, USA) (0.5 mg/mL). The MTT formazan precipitate was dissolved in 200 μL of dimethyl sulfoxide (DMSO) measured by ELISA reader at 570nm.

### Antibodies and drugs

The antibodies: anti-cofilin-1 (sc-53934), anti-Bcl-2 (sc-7382), anti-β-actin (sc-47778), and anti-p-Akt1/2/3 (sc-7985) were purchased from Santa Cruz (CA, USA), anti-c-caspase-3 (#9664) were purchased from Cell Signaling Technology (Danvers, USA). All the secondary antibodies (horseradish peroxidase-conjugated) were purchased from Santa Cruz. All of the chemo reagents were purchased from Sigma-Aldrich Company.

### Western blot analysis

Total protein from cell samples was measured by Bradford protein assay dye (Bio-Rad, USA). According to our previous study [47], the samples (20 μg) were separated by SDS-PAGE and then transferred to polyvinylidene fluoride membranes (Millipore, Belford, Massachusetts, USA) by the Bio-Rad western blotting system. Briefly, the membranes were blocked for 1 hr with blocking buffer then incubated with specific primary antibodies (dilution 1:1000) at 4°C overnight. Following, the membrane will conjugate with secondary antibodies for 1 h then incubated with chemiluminescence buffer (Millipore, Billerica, MA, USA) to obtain images by GE Digital Imaging System (Commerce, CA, USA).

### RNA preparation and quantitative real-time PCR analysis

Total RNA isolation and miRNA analysis was followed our previous study [48] by the RNA Isolation kit (Zymo Research, Irvine, CA, USA) and cDNA was synthesized from sample RNA. The cDNA (1 μg) were used with appropriate primer to perform real-time PCR by SYBR Green buffer (Bio-Rad). The primer sequence of miR-107: AGCAGCATTGTACAGGGCTATCA. mRQ and U6 primer were obtained from the Mir-X miRNA Synthesis Kit.

### Microarray array assay

Total RNA (2 μg; OD260/OD280 = 2.01) was extracted from liver cancer cell lines and sent to miRNA Microarray Services (Human miRNA OneArray, Phalanx Biotech, Hsinchu, Taiwan). The fold change was calculated according to our previous method [48].

### TUNEL assay

The tissue sections (2-μm thick) were stained with the UNEL Assay reagent (Roche Ltd., Basel, Switzerland), the cell nuclei will stain by DAPI (4′,6-diamidino-2-phenylindole; Sigma-Aldrich, Inc.). After that, the tissue sections were obtained the signal by fluorescence microscope (Olympus, Tokyo, Japan).

### MiR-107 upregulation (mimic) or knockdown (inhibitor) by transfection

The miR-107 mimic and inhibitor were purchased from Phalanx Biotech, Hsinchu, Taiwan. After cells were grown to 60%–70% confluent were transfected with the mimic or inhibitor by jetPRIME transfection reagent (Illkirch, France) transfection reagent. After 24 h, the cells were used for subsequent experiments or analysis.

### Luciferase assay

Luciferase reporter assays was carried out as previously described [48]. The miR-107 target expression vector contained with the wild-type cofilin-1 3' UTR sequence or empty. Each plasmid was transfected into cells with concentration of 1 μg/mL for 24 hr.

### Animal model

According to our recent research [21, 48], we obtained the male NU/NU mice (six-week-old) from BioLASCO Taiwan (Taipei, Taiwan). The animal experiment protocol will follow the China Medical University Institutional Animal Care and Use Committee of guidelines No.2018-312. The mice were randomly divided for four groups (*n* = 4): parental and resistance cells control groups (subcutaneous injection of cancer cell HA22T and HDACi-R cells, two tumors in each mice), miR-107-treated HA22T, and HDACi-R groups. Briefly, cancer cells (1 × 10^7^ cells) will mix with Matrigel matrix and 50% serum-free DMEM. The miR-107 agomir 15 nM (Phalanx Biotech, Hsinchu, Taiwan) was delivered by intratumoral injection on the 21^st^ and 28^th^ day after subcutaneous injection. After sacrificed, the tissue was fixed using 10% formaldehyde or stored at -80°C for further analysis.

### Statistical analysis

All cell experiments were independently repeated three times. Statistical analysis was using one-way analysis of variance (ANOVA; Student’s *t*-test) with SigmaPlot 10.0 software (Systat Software Inc., San Jose, CA, USA). ^*^ and ^#^*P* < 0.05 were considered statistically significant and ^**^ and ^##^*P* < 0.01 or ^***^ and ^###^*P* < 0.001 were considered to indicate increased statistical significance. ^*^ Indicates comparison with HA22T control group and ^#^ indicates comparison with HDACi-R control group.
